# Magnetic resonance imaging of the proximal tibial epiphysis is suitable for statements as to the question of majority: a validation study in forensic age diagnostics

**DOI:** 10.1007/s00414-021-02766-x

**Published:** 2021-12-28

**Authors:** Daniel Wittschieber, Natia Chitavishvili, Ismini Papageorgiou, Ansgar Malich, Gita Mall, Hans-Joachim Mentzel

**Affiliations:** 1 Institute of Legal Medicine, Jena University Hospital, Friedrich Schiller University Jena, Am Klinikum 1, 07747 Jena, Germany; 2 Section of Pediatric Radiology, Department of Radiology, Jena University Hospital, Friedrich Schiller University Jena, Am Klinikum 1, 07747 Jena, Germany; 3 Institute of Diagnostic and Interventional Radiology, Jena University Hospital, Am Klinikum 1, 07747 Jena, Germany; 4 Institute of Radiology, Südharz Klinikum Nordhausen, Dr.-Robert-Koch-Straße 39, 99734 Nordhausen, Germany

**Keywords:** Forensic age estimation, Knee joint, Radiation-free, Age of majority, Vieth’s classification system

## Abstract

Determining majority plays a key role for forensic age diagnostics in living individuals. Recent data suggest that magnetic resonance imaging (MRI) of the proximal tibial epiphysis (PTE) may be a suitable alternative or at least an additional tool to clarify whether an individual has reached majority. However, the reference data situation is still sparse. Hence, the present dual center study retrospectively analyzed routine MRI of the knee in 413 cases (214 males and 199 females) of a Western Caucasian population aged between 12 and 25 years. MRI was performed at 1.5 and 3.0 T clinical scanners using T1- and T2-weighted sequences. The classification system by Vieth et al. (Eur Radiol 2018; 28:3255–3262) was applied for determining the ossification stages of the PTE. Intra-observer agreement was “very good” (*κ* = 0.931), and inter-observer agreement was “good” (*κ* = 0.798). Minimum ages above the age of 18 years were observed with the final stage (stage 6) in either sex (20.27 years in males and 18.55 years in females). The results are not in contradiction with the previous data and can be considered a strong and valuable support of the so far existing database. Therefore, the investigation of the PTE using routine MRI (either at 1.5 or 3.0 T) could be taken into consideration for application in forensic age estimation practice in near future.

## Introduction

Given growing cross-border migration in the last decade, forensic age estimations have been increasingly requested by courts and other government authorities across Europe in order to proof whether people without valid identification documents have exceeded legally defined age thresholds [1]. In civil and criminal law of most European countries, the age of 18 years is regarded as the age of majority; therefore, the completion of this age plays a key role for numerous legal decisions [2]. In those cases, the Study Group on Forensic Age Diagnostics (AGFAD) of the German Society of Legal Medicine (DGRM) still recommends an additional evaluation of the ossification status of the medial clavicular epiphysis [3], either by projection radiography, which should be considered obsolete today [4], or by computed tomography. The latter has been well established in European age estimation practice over time [2, 5–8].

However, both procedures are related to ionizing radiation, which is why these X-ray examinations usually require a legal basis for authorization [2]. Hence, for age estimations without legitimation for X-ray examinations, radiation-free imaging methods such as ultrasound or magnetic resonance imaging (MRI) would be desirable to determine skeletal age. Although the general feasibility of MRI in forensic age diagnostics has repeatedly been shown for several maturation indicators [9–14], MRI has not been implemented into novel AGFAD recommendations yet. This is possibly attributed to the facts that, on the one hand, reference data meeting the criteria defined by the AGFAD [3] are still sparse, and, on the other hand, that the application of established classification systems on initially promising body regions using MRI [15] finally turned out to be unsuitable for determining majority [16, 17]. However, a recent study by Vieth et al. (2018) [18], presenting a novel classification system based on information from both T1-weighted and T2-weighted MR sequences, suggests that MRI of the knee joint-associated epiphyses may be a suitable alternative or at least an additional tool to clarify whether an individual has reached majority. Due to its novelty, the reference data situation concerning this novel classification system is inherently poor.

Therefore, the present study investigates the age-dependent epiphyseal ossification process of the proximal tibial epiphysis (PTE) using MRI and the novel classification system by Vieth et al. [18]. The primary objective is the evaluation and validation of the novel classification system, especially concerning its practicability in forensic age estimation practice, and as to the question of whether MRI data from routine diagnostics can be employed as well. Furthermore, the enlargement of the general database is aimed for possible practical application of the novel method in near future.

## Materials and methods

The local ethics committee of the Jena University Hospital (reference number “2019–1362-Daten”) as well as the ethics committee of the medical association of the Federal State of Thuringia (reference number “53,394/2019/129”) approved the study.

MRI scans of the knee joint generated mainly for traumatological indications between 2010 and 2019 at the Institute of Diagnostic and Interventional Radiology of the Jena University Hospital as well as at the Südharz Klinikum Nordhausen (Academic Teaching Hospital of the Jena University Hospital) were evaluated retrospectively (*n* = 442). No difference was made between left and right side. The subjects had to be aged between 12 and 25 years at the time of the MRI scan, and the corresponding medical records were not allowed to reveal any disease affecting the skeletal development or injuries or post-surgical lesions of the knee. Age and sex distribution had to be as even as possible. Image quality was assessed using a Likert scale from 1 to 3 (1 = poor, 2 = good, 3 = excellent quality). During the later evaluation, 29 cases were excluded due to poor image quality (Likert 1, *n* = 5), or due to different artifacts impeding the determination of the ossification stage (*n* = 24), e.g., movement artifacts or superimpositions by bone marrow edema.

The final study cohort consisted of 413 assessable cases (214 males and 199 females) (Table 1). According to the corresponding medical records (names, places of birth) and according to the general population structure of the Federal State of Thuringia (Central Germany), the vast majority of the study subjects can be considered part of a Western Caucasian population. Therefore, a high socio-economic status of the study cohort can be assumed.

**Table 1:** Number of assessable cases by age and sex (*n* = 413)

Age group (years)	Males	Females
12	13	15
13	18	17
14	16	12
15	12	17
16	16	13
17	15	13
18	16	15
19	16	14
20	17	15
21	14	16
22	18	18
23	15	15
24	16	11
25	12	8
Σ	214	199

All MRI scans were performed according to standard procedures at various 1.5 T or 3.0 T MR scanners (Siemens, Erlangen, Germany; Philips, Eindhoven, The Netherlands). Due to the retrospective study design, no additional protocols were applied. For the determination of the ossification stage, coronal views were considered only. Each case evaluation was performed using two different sequences as follows (in brackets: first, the data for 1.5 T, and after slash the data for 3.0 T):

- a T1-weighted turbo spin echo sequence (T1w TSE; TR 540/750 ms; TE 7.3/19 ms; flip angle 90/120°; field of view 160 mm; slice thickness 3.0 mm) and
- a proton-density-weighted turbo spin echo sequence with fat suppression (PD TSE FS) (TR 3390/4720 ms; TE 30/39 ms; flip angle 90°; field of view 160 mm; slice thickness 3.0 mm) or
- a T2-weighted turbo-inversion recovery-magnitude sequence (TIRM) (TR 3720/3770 ms; TE 32/80 ms; flip angle 173/143°; field of view 160 mm; slice thickness 3.0 mm).

**Fig. 1:**
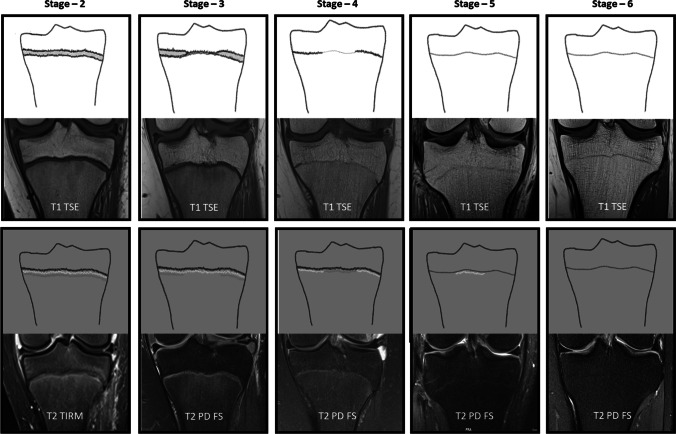
The classification system by Vieth et al. [18], schematic drawings and case examples from our study cohort

The image material was evaluated using a standard PACS workstation and certified monitors. To assess the degree of the ossification of the proximal tibial epiphysis, the classification system by Vieth et al. [18] was applied (Table 2, Fig. 1). The assessments were done consensually by three readers: a pediatric radiologist (reader 1), and a forensic physician (reader 2), each of which with more than 10 years of specific experience in forensic age diagnostics using skeletal imaging, and a trainee and doctoral candidate in pediatric radiology (reader 3). For the evaluation of the intra- and inter-observer agreement, 100 cases were randomly chosen and re-assessed by reader 1 and 3. To avoid a recall bias, the re-assessments were done two months after the consensual assessment (reader 1 and 3) and again two months after the first re-assessment (reader 3 alone), respectively. The readers were blinded to the age and sex of the individuals before and during the evaluation process.

**Table 2:** Original descriptions of the ossification stages defined by Vieth et al. [18]. Bold text highlights relevant differences between the stages

Stage	Type of sequence	Original descriptions
Stage 2	T1	A continuous band of intermediate signal intensity is visible, walled by serrated lines of low to no signal intensity towards the epiphysis and the diaphysis
	T2	The epiphysis is demarked by a serrated line of low to no signal intensity. The metaphysis shows two serrated lines of high signal intensity. Both lines can be continuous or discontinuous
Stage 3	T1	A discontinuous band of intermediate signal intensity is visible. The band is walled by serrated lines of low to **no signal intensity towards the epiphysis and the diaphysis that sporadically convene and interrupt the band, forming a single serrated line with no signal intensity**
	T2	The metaphysis shows two serrated lines of **high signal intensity that sporadically convene, forming a single thin and serrated line of high signal intensity**
Stage 4	T1	A discontinuous thin and serrated line of intermediate signal intensity between the epiphysis and the diaphysis is visible. In the continuity of the line, **thicker sections with no signal intensity** can be seen
	T2	A thin single, discontinuous or dotted line of hyperintense signal is visible in the same position as the described thin line of the corresponding T1-w sequence. In the continuity of the line, **thicker hyperintense sections** can be seen
Stage 5	T1	A continuous thin line of intermediate signal intensity between the epiphysis and the diaphysis is visible
	T2	A single thin, discontinuous or dotted line of **hyperintense signal** in the same position as the described thin line of the corresponding T1-w sequence
Stage 6	T1	A continuous thin line of intermediate signal intensity between the epiphysis and the diaphysis is visible
	T2	**No hyperintense signal** in the same position as the described thin line of the corresponding T1-w sequence

Statistical analyses were performed using IBM SPSS Statistics Version 27 (release 17 June 2020). Age data obtained for each ossification stage are expressed as minimum, maximum, mean ± standard deviation, and median with lower and upper quartiles. Sex-related differences were analyzed by Mann–Whitney U test for two independent groups. The influence of the image quality (Likert scale) on stage determination was tested using Spearman’s rank correlation (r_s_). *P* < 0.05 (exact, two-sided) was considered statistically significant. Intra- and inter-observer agreements were determined by means of Cohen’s kappa (κ) non-parametric test. For the interpretation of κ values, the system proposed by Altman was used [19]: *κ* < 0.20, poor agreement; *κ* = 0.21–0.40, fair agreement; *κ* = 0.41–0.60, moderate agreement; *κ* = 0.61–0.80, good agreement; *κ* = 0.81–1.00, very good agreement.

## Results

The MR image quality (assessed on a Likert scale from 1 to 3) did not show a significant influence on the process of stage determination (Spearman correlation coefficient *r*_*s*_ = 0.17, *p* = 0.092, and repetition after 2 months with the same patients, Spearman correlation coefficient *r*_*s*_ = 0.105, *p* = 0.299).

**Fig. 2:**
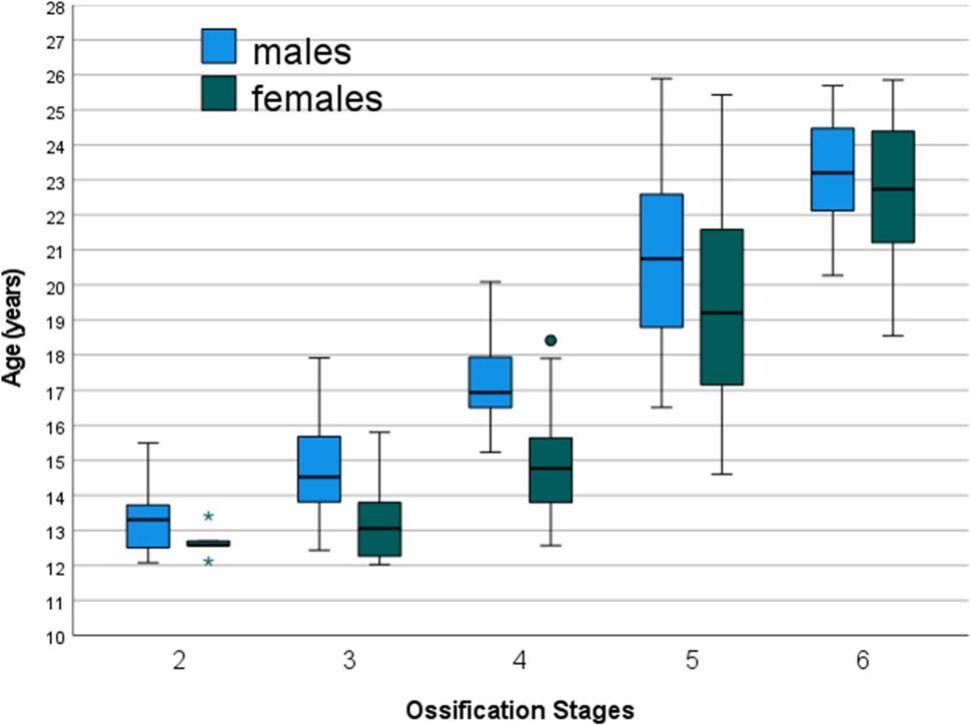
Box-and-whisker plot diagram visualizing the age ranges obtained for all ossification stages determined. Both sexes are presented separately. The whiskers show the minimum and maximum ages

Figure 2 presents a box-and-whisker plot diagram visualizing the age ranges obtained for all ossification stages determined. On the one hand, constantly increasing age medians (black horizontal bars within the boxes) can be recognized from stage to stage and for both sexes, suggesting a good discrimination between the ossification stages of the staging system and, thereby, capturing the ossification and fusion process of the PTE as expected. On the other hand, an accelerated development of the males can be assumed. Accordingly, as shown in Table 3, sex-related differences were found to be statistically significant in stages 3 (*p* < 0.001), 4 (*p* < 0.001), and 5 (*p* = 0.001). Furthermore, a relatively large scatter is striking for stage 5 in both sexes.

**Table 3:** Synopsis of the statistical parameters expressed in years (*n* = 413). Note that in both sexes the minimum ages of the stages 2 and 3 as well as the maximum ages of the stages 5 and 6 must not be used in age assessment practice due to truncation of the age interval of the study population

Stage	Sex	*n*	Minimum	Maximum	Mean ± SD	LQ	M	UQ	*p* (male vs. female)
2	Male	26	12.08	15.50	13.33 ± 0.94	12.50	13.30	13.78	0.217
	Female	5	12.11	13.41	12.67 ± 0.47	12.34	12.57	13.05	
3	Male	36	12.43	17.92	14.69 ± 1.37	13.76	14.52	15.77	< 0.001 *
	Female	17	12.03	15.80	13.21 ± 1.03	12.23	13.06	13.80	
4	Male	24	15.23	20.08	17.28 ± 1.31	16.46	16.94	18.01	< 0.001 *
	Female	33	12.57	18.41	14.94 ± 1.58	13.79	14.77	15.93	
5	Male	84	16.52	25.89	20.84 ± 2.53	18.73	20.74	22.61	0.001 *
	Female	96	14.61	25.43	19.41 ± 2.88	17.16	19.21	21.59	
6	Male	44	20.27	25.69	23.17 ± 1.49	22.12	23.20	24.52	0.298
	Female	48	18.55	25.86	22.69 ± 1.97	21.03	22.74	24.54	

*Minimum* = minimum age, *Maximum* = maximum age, *SD* = standard deviation, *LQ* = lower quartile, *M* = median, *UQ* = upper quartile * = statistically significant

Table 3 shows the synopsis of the detailed statistical parameters. In males, the stages 2, 3, 4, 5 and 6 were first observed at the minimum ages of 12.08, 12.43, 15.23, 16.52, and 20.27 years, respectively. In females, the stages 2, 3, 4, 5 and 6 were first observed at the minimum ages of 12.11, 12.03, 12.57, 14.61, and 18.55 years respectively.

Regarding the repeatability and reproducibility of the staging method applied in this study, intra- and inter-observer agreements were calculated using κ statistics. When comparing the 100 stage determinations by reader 1 (first re-assessment) with those by reader 1 (second re-assessment), the intra-observer agreement corresponded to a “very good agreement” (*κ* = 0.931). When comparing the 100 stage determinations by reader 1 with those by reader 3, the inter-observer agreement was found to be a “good agreement” (*κ* = 0.798). The comparisons performed for intra- and inter-observer agreements also revealed that, if any, most different stage determinations occurred concerning the question of stage 5 or 6.

## Discussion

The present study confirmed that the novel classification system by Vieth et al. [18] can also be applied to MRI data from routine diagnostics using both 1.5 T and 3.0 T scanners as well as other T2 sequences than the T2-TSE SPIR sequence used in the original study. However, it has to be emphasized that T2 sequences *with fat saturation* are necessary in order to determine Vieth stages. Our observations are in line with two other recent studies that also retrospectively applied the classification system by Vieth et al. [18] to MRI of the knee joint in two Turkish study populations [20, 21]. As also stated by Saint-Martin et al. (2015) [22] as well as by Gurses and Altinsoy (2020) [20], we likewise obtained similar results with MR image material from 1.5 T and 3.0 T scanners, and we were able to identify all relevant structures required for stage determinations despite the fact that T2-TSE SPIR sequences were not available.

Table 4 shows a comparison of the so far existing studies investigating MRI of the PTE using the classification system by Vieth et al. [18]. The data of the present study corroborated that, at least in male individuals, MRI of the PTE is suitable to determine majority when using the classification system by Vieth et al. [18]. Although we observed minimum ages for stage 6 above the age of 18 years in both sexes (20.27 years in males and 18.55 years in females), we are aware that, especially with regard to forensic age estimation practice, both the original study by Vieth et al. [18] and the two above-mentioned Turkish studies [20, 21] have already found minimum ages for stage 6 in females below the age of 18 years. Thus, our results are not in contradiction with the previous data. However, our data can only be considered a strong and valuable support of the more comprehensive and prospectively collected age data presented by Vieth et al. [18].

**Table 4:** Comparison of the studies investigating MRI of the PTE using the classification system by Vieth et al. [18]

	Vieth et al. (2018) [18]	Gurses et al. (2020) [20]	Alatas et al. (2021) [21]	Present study
General characteristics				
Case number	694	598	709	413
Study design	prospective	retrospective	retrospective	retrospective
Age groups considered [years]	12–24	12–30	12–27	12–25
Geographic origin of the study cohort	Germany	Turkey	Turkey	Germany
HDI rank of the country	6	54	54	6
Technical parameters				
Field strength(s)	3.0 T	1.5 T	1.5 T	1.5 T / 3.0 T
T1w sequence	TSE (coronal)	TSE (sagittal/coronal)	TSE (coronal)	TSE (coronal)
Slice thickness (T1)	3.0 mm	3.5 mm	3.5 mm	3.0 mm
T2w sequence(s)	TSE SPIR (coronal)	FS PD TSE (coronal)	PD SPAIR TSE (coronal)	PD TSE FS or TIRM (coronal)
Slice thickness (T2)	3.0 mm	3.0 mm	3.5 mm	3.0 mm
Minimum ages of the ossification stages [years]				
Stage 2 (males)	12.05	12.08	12.02	12.08
Stage 2 (females)	12.56	12.08	12.01	12.11
Stage 3 (males)	12.13	12.25	12.07	12.43
Stage 3 (females)	12.11	12.50	12.01	12.03
Stage 4 (males)	14.68	13.83	13.57	15.23
Stage 4 (females)	12.48	13.08	13.24	12.57
Stage 5 (males)	15.71	15.58	15.59	16.52
Stage 5 (females)	14.44	14.75	14.38	14.61
Stage 6 (males)	19.85	18.83	18.91	20.27
Stage 6 (females)	17.65	17.25	16.87	18.55

*HDI* = human development index 2020 [23], *T1w* = T1 weighted, *T2w* = T2 weighted, *TSE* = turbo spin echo, *SPIR* = spectral pre-saturation with inversion recovery, *FS* = fat suppressed, *PD* = proton density, *SPAIR* = spectral attenuated inversion recovery

This result reveals the most relevant limitation of our study: the retrospective study design. Despite the acquisition of retrospective knee MRI data from two large tertiary care hospitals of a 10-year period, we were able to include 413 cases only, which is considerably lower than the 694 cases of the original study by Vieth et al. [18]. The lower case number may therefore explain not only the lack of female individuals below the age of 18 with a PTE showing stage 6, but also the comparably higher minimum age in males concerning stage 6.

Besides the limitations, the present study has also some noteworthy strengths. Unlike the other two subsequent studies from Turkey [20, 21], which both stated a lack of information on the socio-economic status of their study patients, a high socio-economic status of the present Central German study cohort can be assumed because the vast majority of the subjects can be considered part of a Western Caucasian population. This is of great relevance because, as also stated by Vieth et al. [18], it is well known from earlier studies [24, 25] that the Western Caucasian ethnicity with high socio-economic status displays the fastest progression of skeletal maturation. Hence, the application of the minimum ages of a reference study investigating a population with high socio-economic status, which are the most relevant when using the so-called “minimum age concept” (e.g., in criminal proceedings) [2], will rather lead to an underestimation of age when applied to other ethnicities, particularly to those with lower socio-economic status.

Another strength is the relatively even distribution of our study subjects across age groups and sexes, which was not the case in the two Turkish studies [20, 21]. However, both an even age distribution and data on the reference population (regarding genetic/geographic origin) and socio-economic status, respectively, belong to the AGFAD criteria required for reference studies in forensic age estimation [3].

MRI of the PTE has already been investigated for the purpose of forensic age diagnostics by several other authors with some other staging systems. As early as in 2010, Jopp et al. [26] studied a small pilot sample of 41 young males between 15 and 19 years using 1.5 and 3.0 T MRI and a 3-category system based on the epiphyseal-diaphyseal osseous fusion of the PTE. In 2012, Dedouit et al. [10] used 290 MRI scans of patients aged between 10 and 30 years and introduced a 5-stage system for evaluating MRI of both knee epiphyses based on the absolute measureable thickness of growth plate layers. However, although the presence of a stage 5 is possibly able to determine age of majority [10, 27], the general approach of the staging system has been criticized as the physical dimensions of anatomical structures are associated with the individual’s body height and might therefore be unsuited for absolute measurements [18]. Later studies applied the classical staging system composed of 5 main stages according to Schmeling et al. [28] and 6 sub-stages according to Kellinghaus et al. [29] to T1-weighted (closest-to-bone) MRI sequences of both knee epiphyses [16, 17]. However, this approach finally did not facilitate the determination of majority in either sex. Concerning this matter, Vieth et al. [18] pointed out that the Schmeling/Kellinghaus system does not take into account the watery components and soft tissues of the osseous structures.

Hence, the staging system by Vieth et al. [18] aimed at considering more aspects of the morphological appearance of the epiphyseal-diaphyseal fusion. Although, as also stated by the authors themselves [18], the relevant landmarks of each stage are mainly drawn from the T1-weighted sequence, stage 5 and 6, which are crucial for the question of majority, have exactly the same appearance in T1 and absolutely require information from the T2-weighted sequence. Recognizing the faint hyperintense signal, defining the difference between stage 5 and 6, also represented a very small, but the most striking difficulty in our study. Possibly, this may be due to its relative novelty. Moreover, it still remains an interesting question what is the origin of this hyperintense signal. It is known, that T2 changes within the bone are much more sensitive to remodeling processes than T1 signal. For example, edema can be identified much longer in the event of trauma or inflammation. This could also apply to modeling processes in the epiphyseal areas.

In the present study, cases with different ossification stages in T1 and T2 only occurred very rarely. In order to merge the information of T1 and T2 into a single ossification stage, the rules previously developed for the combination of multiple CT slices of the medial clavicular epiphysis [6, 30] were modified as follows: stage 2 (in one sequence) + stage 3 (in the other sequence)→stage 3, stage 3 + stage 4→stage 4, stage 4 + stage 5→ stage 4, stages 5 and 6 can only be distinguished by T2 [18].

Although the staging system by Vieth et al. [18] was originally developed for both knee epiphyses, it may have potential for other growth plates as well. Recently, it has already been applied to the proximal humeral epiphysis [31]. The authors investigated a study population of 315 individuals and found minimum ages in either sex above the age of 18 years for both stage 5 (19.32 in males, 19.20 in females) and stage 6 (22.21 in males, 22.19 in males). Future studies will show whether the approach is able to prevail.

## Conclusions

The present study corroborated the suitability of the statistical data for the PTE shown in the original study by Vieth et al. [18]. Furthermore, our data are also in line with the first subsequent studies from Turkey [20, 21]. In male individuals, which by far account for the most frequent group in forensic age estimation practice, the age of majority can be determined when stage 6 is present. Therefore, the investigation of the PTE using routine MRI (either at 1.5 or 3.0 T) could be taken into consideration for application in forensic age estimation practice in near future; either as additional indicator of maturation only, or at least in cases when CT of the medial clavicular epiphysis revealed non-assessable image material (e.g., anatomical shape variants on both sides). Moreover, a simultaneous and continuous enlargement of the database is still necessary.
